# Identification of IQM-266, a Novel DREAM Ligand That Modulates K_V_4 Currents

**DOI:** 10.3389/fnmol.2019.00011

**Published:** 2019-02-04

**Authors:** Diego A. Peraza, Pilar Cercós, Pablo Miaja, Yaiza G. Merinero, Laura Lagartera, Paula G. Socuéllamos, Carolina Izquierdo García, Sara A. Sánchez, Alejandro López-Hurtado, Mercedes Martín-Martínez, Luis A. Olivos-Oré, José R. Naranjo, Antonio R. Artalejo, Marta Gutiérrez-Rodríguez, Carmen Valenzuela

**Affiliations:** ^1^Instituto de Investigaciones Biomédicas Alberto Sols (IIBM), CSIC-UAM, Madrid, Spain; ^2^Spanish Network for Biomedical Research in Cardiovascular Research (CIBERCV), Instituto de Salud Carlos III, Madrid, Spain; ^3^Instituto de Química Médica (IQM), IQM-CSIC, Madrid, Spain; ^4^Instituto Universitario de Investigación en Neuroquímica & Departamento de Farmacología y Toxicología, Facultad de Veterinaria, UCM, Madrid, Spain; ^5^Centro Nacional de Biotecnología (CNB), CNB-CSIC, Madrid, Spain; ^6^Spanish Network for Biomedical Research in Neurodegenerative Diseases (CIBERNED), Instituto de Salud Carlos III, Madrid, Spain

**Keywords:** K_V_4.3 channels, DREAM, DREAM ligands, KChIP, A-type current, Alzheimer

## Abstract

Downstream Regulatory Element Antagonist Modulator (DREAM)/KChIP3/calsenilin is a neuronal calcium sensor (NCS) with multiple functions, including the regulation of A-type outward potassium currents (*I*_A_). This effect is mediated by the interaction between DREAM and K_V_4 potassium channels and it has been shown that small molecules that bind to DREAM modify channel function. A-type outward potassium current (*I*_A_) is responsible of the fast repolarization of neuron action potentials and frequency of firing. Using surface plasmon resonance (SPR) assays and electrophysiological recordings of K_V_4.3/DREAM channels, we have identified IQM-266 as a DREAM ligand. IQM-266 inhibited the K_V_4.3/DREAM current in a concentration-, voltage-, and time-dependent-manner. By decreasing the peak current and slowing the inactivation kinetics, IQM-266 led to an increase in the transmembrane charge (${\text{Q}}_{{\text{K}}_{\text{V}}4.3/\text{DREAM}}$) at a certain range of concentrations. The slowing of the recovery process and the increase of the inactivation from the closed-state inactivation degree are consistent with a preferential binding of IQM-266 to a pre-activated closed state of K_V_4.3/DREAM channels. Finally, in rat dorsal root ganglion neurons, IQM-266 inhibited the peak amplitude and slowed the inactivation of *I*_A_. Overall, the results presented here identify IQM-266 as a new chemical tool that might allow a better understanding of DREAM physiological role as well as modulation of neuronal *I*_A_ in pathological processes.

## Introduction

The Downstream Regulatory Element Antagonist Modulator (DREAM; Carrion et al., 1999), also known as KChIP3 (An et al., 2000) or calsenilin (Buxbaum et al., 1998), is a member of the K_V_ channel interacting proteins (KChIPs) belonging to the neuronal calcium sensor (NCS) family (Burgoyne, 2007). DREAM is a 29 kDa protein with four EF-hand motif (EF1–4), conserved among other NCS members, in which the EF2 mediates low affinity Ca^2+^ binding and is occupied by Mg^2+^ under physiological conditions, whereas EF3 and EF4 mediate high affinity Ca^2+^ binding (Bahring, 2018). The physiological roles of DREAM have been gradually revealed. In the nucleus, DREAM binds to a specific DRE to repress transcription of target genes (Carrion et al., 1999; Cheng et al., 2002; Ruiz-Gomez et al., 2007; Wu et al., 2010). Outside the nucleus, DREAM interacts with presenilins to modulate calcium release from the endoplasmic reticulum (Lilliehook et al., 2002). Additionally, the role of the downregulation of DREAM as part of an endogenous neuroprotective mechanism that improves ATF6 processing, neuronal survival in the striatum, and motor coordination in R6/2 mice, a model of Huntington’s disease (HD), has been recently described (Naranjo et al., 2016; López-Hurtado et al., 2018). Besides, DREAM acts as a regulatory subunit of K_V_4.3 channels by inducing: (i) increased traffic of K_V_4.3 channels to the membrane; (ii) delayed inactivation kinetics; and (iii) accelerated activation and recovery kinetics from inactivation of K_V_4.3 channels (An et al., 2000). K_V_4 α-subunits, KChIPs and dipeptidyl aminopeptidase-like proteins (DPPs) form ternary complexes, which regulate the A-type outward potassium currents in neurons (*I*_A;_ Nadal et al., 2003; Maffie and Rudy, 2008). *I*_A_ is responsible for the fast repolarization of neuron action potentials and the frequency of firing, and thereby controls neuronal excitability (Birnbaum et al., 2004; Johnston et al., 2010). Among the K_V_4 α-subunits, K_V_4.2 and K_V_4.3 underlie the somatodendritic *I*_A_ in the central nervous system (CNS; Huang et al., 2005), whereas, K_V_4.1 mRNA levels are lower than K_V_4.2 or K_V_4.3 (Serôdio and Rudy, 1998). Among other KChIPs, DREAM binding to K_V_4 channels regulates potassium currents, and hence neuronal excitability, in response to changes in intracellular calcium. Alterations in the function of the complexes K_V_4/KChIP and/or DREAM are implicated in different neuronal pathologies such as Alzheimer’s (Hall et al., 2015) and HD (Naranjo et al., 2016), spinocerebellar ataxia (Smets et al., 2015) or epilepsy (Villa and Combi, 2016). Additionally, small molecules that bind to DREAM also modify channel function (Gonzalez et al., 2014; Naranjo et al., 2016). In this regard, repaglinide and CL-888 showed an inhibition of the *I*_A_ (Naranjo et al., 2016), whereas NS5806 is the only described DREAM ligand showing a potentiation of *I*_A_ under certain conditions (Witzel et al., 2012). Hence, it would be of great interest to have broader range of chemical tools that might allow a better understanding of the physiological role of DREAM and the modulation of neuron *I*_A_ in pathological processes.

In this work, using surface plasmon resonance (SPR) assays and electrophysiological recordings of K_V_4.3/DREAM channels, we described IQM-266 as a new DREAM ligand able to inhibit the K_V_4.3/DREAM current in a concentration- and voltage-dependent manner, and to slow the activation and the inactivation kinetics. Blocking the peak current and slowing the inactivation kinetics led to an increase in the transmembrane charge (${\text{Q}}_{{\text{K}}_{\text{V}}4.3/\text{DREAM}}$) at a certain range of concentrations, identifying IQM-266 as a new activator of the DREAM-mediated K_V_4.3 currents. Importantly, *I*_A_ recording from rat dorsal root ganglia (DRG) neurons revealed IQM-266 effects reminiscent of those observed in K_V_4.3/DREAM channels. Our findings offer new possibilities to control neuronal hyperexcitability by modulating the potassium outward current through K_V_4 channel complexes.

## Materials and Methods

All experiments shown in the present study were performed through the NIH rules (Guide for the care and use of laboratory animals; NIH publications number 23-80) revised in 2011; as well as the European Parliament 2010/63/EU and the rules of the Helsinki Declaration.

### IQM-266 Chemical Synthesis

3-(2-(3-Phenoxyphenyl)acetamido)-2-naphthoic acid: 2-(3-phenoxyphenyl)acetic acid (1.5 equiv.) in SOCl_2_ (2 mL/mmol) was refluxed for 6 h, and the excess of thionyl chloride was evaporated to dryness. The residue was then dissolved in anhydrous THF (2 mL/mmol), and 3-amino-2-naphthoic acid (1.0 equiv.) and propylene oxide (15.0 equiv.) were added to the solution. After stirring overnight at room temperature, the solvent was evaporated to dryness and the crude residue was dissolved in EtOAc (3 × 10 mL), washed with brine (30 mL) and dried over Na_2_SO_4_. After removal of the solvent to dryness, the residue was triturated with Et_2_O, and the resultant solid subsequently triturated with CH_3_CN. The obtained solid was lyophilized to give a brown pale solid. m.p. 198.1–196.3°C. **^1^H-NMR** (400 MHz, dimethyl sulfoxide (DMSO)-*d_6_*) δ (ppm): 3.81 (s, 2H, CH_2_CO), 6.94 (ddd, *J* = 8.2, 2.5, 0.9 Hz, 1H, H_4’_), 7.03 (dt, *J* = 7.7, 1.1 Hz, 2H, H_2”, 6”_), 7.07 (t, *J* = 2.5 Hz, 1H, H_2’_), 7.11 (tt, *J* = 7.7, 1.1 Hz, 1H, H_4”_), 7.17 (dt, *J* = 8.2, 0.9 Hz, 1H, H_6’_), 7.33–7.41 (m, 3H, H_5’, 3”, 5”_), 7.47 (ddd, *J* = 8.1, 6.9, 1.1 Hz, 1H, H_6_), 7.60 (ddd, *J* = 8.1, 6.9, 1.1 Hz, 1H, H_5_), 7.86 (d, *J* = 8.1 Hz, 1H, H_7_), 8.01 (d, *J* = 8.1 Hz, 1H, H_4_), 8.67 (s, 1H, H_8_), 8.94 (s, 1H, H_3_), 11.12 (s, 1H, NH). ^13^**C-NMR** (100 MHz, DMSO-*d*_6_) δ (ppm): 44.3 (CH_2_CO), 116.6 (C_3_), 117.3 (C_1_), 117.4 (C_4’_), 118.5 (C_2”, 6”_), 120.0 (C_2’_), 123.4 (C_4”_), 124.9, 125.6 (C_6’_), 127.1, 128.2, 129.1, 129.3, 130.0 (C_3”, 5”_), 130.2 (C_5’_), 133.1 (C_7a_), 135.5 (C_3a_), 136.0 (C_2_), 137.0 (C_1’_), 156.7 (C_3’, 1”_), 168.6 (CO_2_H), 169.4 (CH_2_CO). **HPLC** (Sunfire C18, gradient 50%–95% of acetonitrile in water, 10 min): t_R_ = 7.04 min. **LC-MS**: 398.2 ([M + H]^+^). **HRMS (EI^+^**) *m/z* found 397.1306 ([M]^+^ C_25_H_19_NO_4_ calculated 397.1314).

### Surface Plasmon Resonance (SPR): Binding Experiments

SPR experiments were performed at room temperature (20°C) with a Biacore X-100 apparatus (Biacore, GE Healthcare Life Sciences) in running buffer (50 mM Tris pH 7.5, 50 mM NaCl, 2 mM CaCl_2_ with 2% DMSO and 0.05% Tween 20). The protein GST-DREAM was immobilized on a CM5 sensor chip (Biacore, GE) following a standard amine coupling method (Johnsson et al., 1991). The carboxymethyl dextran surface of the experimental flow cell was activated with a 7-min injection of a 1:1 ratio of 0.4 M 1-ethyl-3-(3-dimethylaminopropyl)carbodiimide hydrochloride (EDC) and 0.1 M *N*-hydroxysuccinimide. The protein was coupled to the surface with a 7-min injection at several dilutions at 10–100 μg/ml in 10 mM sodium acetate, pH 4.0. Unreacted *N-hydroxysuccinimide* esters were quenched by a 7-min injection of 0.1 M ethanolamine-HCl (pH 8.0). Immobilization levels were in the 7,000–8,000 RUs range. Reference flow cell was treated as experimental flow cell (amine coupling procedure) but without protein. Prior to use, 10 mM stock solutions of IQM-266 compound were diluted several times to a 1–7 μM final concentration in running buffer. Affinity measurements were made by a series of different concentrations injected onto the sensor chip at a 90 μl/min flow rate for 1 min, followed by a 1 min dissociation period. After dissociation, an extra wash was done over the flow cells with 50% DMSO. No regeneration was needed.

Sensograms data were double-referenced and solvent-corrected using the BIAevaluation X-100 software (Biacore, GE Healthcare Life Sciences). Experimental data for affinity measurements were adjusted to a one site-specific model binding with Hill slope, using the equation: $R_{\text{eq}}=R_{\max}{\left[A\right]}^{n}/(K_{\text{D}}^{n}+{\left[A\right]}^{n})$ where *R_eq_* is the equilibrium response at each concentration, *R*_max_ is the maximum specific binding, [A] is the analyte concentration, *K*_D_ the equilibrium dissociation constant and *n* the Hill slope.

### Cellular Cultures and Transient Transfection

All experiments were performed in CHO-K1 (*Chinese Hamster Ovary*, CHO) cells obtained from the American Type Culture Collection (Rockville, MD, USA) and cultured at 37°C in Iscove’s modified Eagle’s medium supplemented with 10% (v/v) fetal bovine serum (FBS), 1% (v/v) L-Glutamine (Gibco), and antibiotics (100 IU/ml penicillin and 100 mg/ml streptomycin; all from Gibco, Paisley, UK) in a 5% CO_2_ atmosphere.

Cells were transiently cotransfected with K_V_4.3 (cloned into pEGFPn1, gently given by Dr. D.J. Synders, University of Antwerpen, Belgium) and DREAM (cloned into pcDNA3.1). In both cases, cells were cotransfected with EBO-pcDLeu2 as a reporter gene, codifying CD8. Transfection was performed using Fugene-6 (Promega) following manufacturer’s instructions as previously described (Moreno et al., 2017; López-Hurtado et al., 2018). After 48 h transfection, cells were removed from culture plates using TrypLE™ Express (Life Technologies), after exposing them to polystyrene microspheres bound to anti-CD8 (Dynabeads M-450, Thermo Fisher Scientific; Franqueza et al., 1999; Naranjo et al., 2016). Because the level of expression of DREAM can be crucial for the effects of IQM-266, only cells cotransfected with K_V_4.3 and DREAM that exhibit a recovery kinetics from inactivation between 20 and 45 ms were selected for electrophysiological recording.

### Electrophysiology

Potassium currents elicited by the activation of K_V_4.3/DREAM channels expressed in CHO cells were recorded at room temperature (20–25°C), at a frequency of 0.1 Hz using the whole-cell patch-clamp technique with an Axopatch 200B patch-clamp amplifier (Molecular Devices) connected to an analogic-digital conversor (Digidata 1322A). Micropipettes were pulled from borosilicate glass capillary tubes (Narishige GD-1) on a programmable horizontal puller (Sutter Instrument Co.) and heat-polished with a microforge (Narishige, Japan). Micropipette resistance was 2–4 MΩ. Data acquisition and genesis of experimental protocols were performed by the CLAMPEX utility of the PCLAMP 9.0.1 program (Molecular Devices). Currents were filtered at 2 kHz and sampled at 4 kHz (Bessel filter of 4 poles). Capacitance and series resistance compensation were optimized, with 80% compensation of the effective access resistance usually obtained. The intracellular pipette filling solution contained (in mM): 80 K-aspartate, 42 KCl, 3 phosphocreatine, 10 KH_2_PO_4_, 3 MgATP, 5 HEPES-K, 5 EGTA-K and it was adjusted to pH 7.25 with KOH. The bath solution contained (in mM): 136 NaCl, 4 KCl, 1.8 CaCl_2_, 1 MgCl_2_, 10 HEPES-Na and 10 glucose and it was adjusted to pH 7.40 with NaOH. IQM-266 was dissolved in DMSO at a stock concentration of 5 mM and added to the external solution at the desired concentration in each experiment. The currents were stored in a computer and analyzed with the CLAMPFIT utility of the PCLAMP 9.0.1 program and Origin 2018 (OriginLab Co.). Origin 2018 (OriginLab Co.) and Clampfit 10 programs were used to perform least-squares fitting and for data presentation (Valenzuela et al., 1994; Longobardo et al., 1998; Naranjo et al., 2016).

In order to obtain the concentration-response curve, block produced by IQM-266 was measured at the maximum peak and under the area of the current after applying different concentrations of the compound (0.01–100 μM) and thus, $\%block=\left(1-\left(\frac{IDrug}{IControl}\right)\right)\times 100$. From the fitting of these values to a Hill equation, concentration-effect curves were generated, obtaining the values of the IC_50_ and the Hill coefficient (*n*).

Activation and inactivation were fitted to a monoexponential process with an equation of the form $y=Ae^{\left(-\frac{t}{\tau }\right)}+C$, where τ represents the system time constant, A represents the amplitude of the exponential, and C is the baseline value. The voltage dependence of the activation curves was fitted with a Boltzmann equation: $y=1/\left(1+e^{\left(-(V-V_{h})/s\right)}\right)$, where *s* represents the slope factor, *V* represents the membrane potential, and *V*_h_ represents the voltage at which 50% of the channels are open. Recovery from inactivativation was analyzed by applying a two pulse protocol consisting in a prepulse from −80 to +60 mV of 1 s in duration, followed by a test pulse to +60 mV of 250 ms in duration after different recovery time. The current measured at the maximum peak and the current in the test pulse were normalized vs. the first prepulse and they were plotted against the recovery time and then fitted to a monoexponential equation in order to obtain the τ_re_ (view “Results” section). In all cases, the control and the experimental condition was the same cell before and after being exposed to IQM-266.

### Isolation of DRG Neurons and Recording of Transient Potassium Currents (*I*_A_)

DRG neurons were isolated from male Sprague-Dawley rats (200–220 g/6–8 weeks old). Rats were sacrificed by cervical dislocation followed by decapitation, and lumbar segments of the spinal column were removed and placed in a cold Ca^2+^, Mg^2+^-free Hank’s solution (Sigma-Aldrich). The bone surrounding the spinal cord was removed and L4, L5 and L6 DRG were exposed and pulled out. After removing the roots, DRG were chopped in half and incubated for 60 min at 37°C in Dulbecco’s modified Eagle’s Medium-low glucose (DMEM; Sigma-Aldrich) containing 5 mg/mL collagenase XI (Worthington Biochemical, Lakewood, NJ, USA), 100 U/ml penicillin (Sigma-Aldrich), and 0.1 mg/ml streptomycin (Sigma-Aldrich). The cell suspension was then washed with DMEM by centrifugation (300 *g*, 5 min at 4°C), filtered through a 100 μm mesh and washed again by centrifugation. The cell pellet was resuspended in DMEM and 40 μl were dropped onto 10 mm diameter glass coverslips treated with poly-D-lysine (1 mg/ml, 30 min; Sigma-Aldrich) and placed in 35 mm diameter Petri dishes. Finally, plated cells were flooded with 2.5 ml of DMEM and supplemented with 10% fetal calf serum (BioWhittaker, UK), 100 U/ml penicillin and 0.1 mg/ml streptomycin, stored in an incubator (Hera Cell, Heraeus, Germany) at 37°C under a 5% CO_2_/95% air atmosphere. This protocol yields spherical cell bodies without neurites, from which only medium DRG neurons (30–40 μm diameter; 30–50 pF) were chosen for recording within 12–24 h of plating.

Current recordings were performed at room temperature (21–24°C) in the perforated-patch variant of the whole-cell configuration of the patch-clamp technique, with an EPC10 amplifier using PatchMaster software (HEKA Electronic, Lambrecht, Germany; Carabelli et al., 2003). Patch pipettes were pulled from borosilicate glass to have a final resistance of 5.5–6.5 MΩ when filled with internal solution (see below). Membrane currents were filtered at 3 kHz and sampled at 10 kHz from cells held at a voltage of −80 mV. Series resistance (<20 MΩ) was compensated by 80% and monitored together with the cell membrane capacitance throughout the experiment. The perforated-patch configuration was obtained using amphotericin B (Sigma-Aldrich) dissolved in DMSO and stored at −20°C in aliquots of 50 mg/ml. The pipette-filling solution contained (mM) 90 K_2_SO_4_, 55 KCl, 8 NaCl, 1 MgCl_2_, 15 HEPES (pH 7.2 with KOH; ≈280 mOsm). Fresh pipette solution was prepared every 2 h. The bath solution containing (mM) 145 NaCl, 2.8 KCl, 2 CaCl_2_, 1 MgCl_2_, 10 HEPES, and 10 glucose (pH 7.4 adjusted with NaOH; ≈300 mOsm) was constantly superfused at a rate of approximately 1 ml/min. IQM-266, at the desired concentration (3 or 10 μM), was directly applied onto the cell under investigation by gravity from a multibarrel concentration-clamp device coupled to electronically driven miniature solenoid valves. The common outlet of this was placed within 100 μm of the cell to be patched.

Transient potassium current (*I*_A_) was isolated by using voltage protocols, taking advantage of the distinct voltage-dependent inactivation of the underlying potassium channels (Phuket and Covarrubias, 2009). First, total voltage-activated potassium current was measured in cells held at −80 mV, in which a 1-s conditioning pulse to −100 mV was delivered prior to 250-ms step depolarizations, ranging from −20 to +20 mV in 10 mV increments. Then, a depolarizing 1-s conditioning pulse to −20 mV was applied, sufficient to inactivate *I*_A_, such that the outward current evoked by subsequent step depolarizations was mostly comprised of a delayed rectifying potassium current. *I*_A_ was finally revealed by subtracting the delayed rectifying current from total current. Peak amplitude of *I*_A_ was used to determine percent block by IQM-266. *I*_A_ inactivation was fitted to a biexponential process with an equation of the form:

$$y=y_{0}+A_{f}\exp\left(\frac{-(x-x_{0})}{\tau_{f}}\right)+A_{s}\exp\left(\frac{-(x-x_{0})}{\tau_{s}}\right)$$

where τ_f_ and τ_s_ represent the fast and slow time constants, respectively, and A_f_ and A_s_ represent the amplitudes of the corresponding kinetic components.

### Statistics

Data are expressed as the mean ± SEM. Direct comparisons between mean values in control conditions and in the presence of drug for a single variable were performed by paired Student’s *t*-test. Differences were considered significant if the *p* value was less than 0.05.

## Results

### Identification of IQM-266 as DREAM Ligand Modulator of K_V_4.3/DREAM Channel Activity

**Figure 1:**
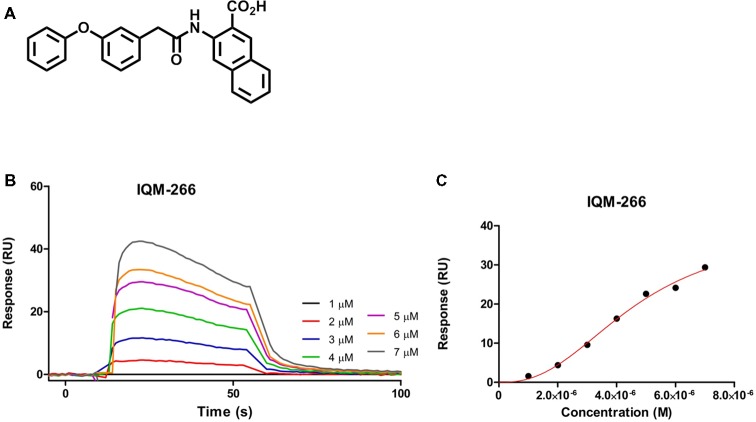
IQM-266 as DREAM ligand. **(A)** Chemical structure of IQM-266. **(B)** Sensograms for binding of IQM-266 using increasing concentrations of the ligand in the range 1–7 μM. RU, resonance units. **(C)** Surface plasmon resonance (SPR) binding plot adjusted with one site specific model with Hill slope.

As part of our program of medicinal chemistry searching for DREAM ligands, we performed a high throughput screening of our chemical libraries to evaluate their binding to DREAM using SPR. We selected the novel compound 3-(2-(3-Phenoxyphenyl)acetamido)-2-naphthoic acid, IQM-266, which showed a *K*_D_ = 4.63 ± 0.73 μM, and we have investigated its effect on the K_V_4/DREAM channels currents (Figure 1; for synthesis and full characterization of IQM-266 see “Materials and Methods” section).

**Figure 2:**
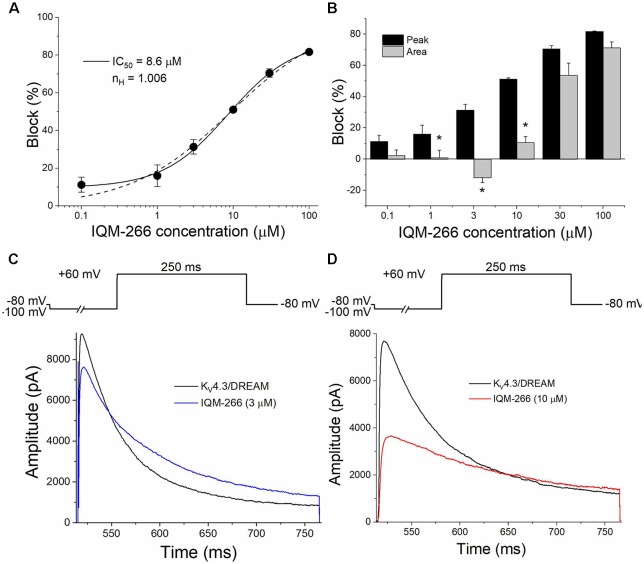
Concentration-dependence of inhibition and/or increase of the current or the charge through K_V_4.3/DREAM channels produced by IQM-266. **(A)** Concentration-response curve of IQM-266 (continuous line). The dashed line represents the fit of the data to a Hill equation with *n*_H_ = 1 (*n* = 34). **(B)** Inhibition or increase of current induced by IQM-266 in K_V_4.3/DREAM channels measured at the maximum peak current and in the charge (measured as the area of the current during the application of a 250 ms pulse to +60 mV; *n* = 34). **(C)** Original recordings obtained after applying a depolarizing pulse to +60 mV in the absence and in the presence of IQM-266 (3 μM). **(D)** Original recordings obtained after applying a depolarizing pulse to +60 mV in the absence and in the presence of IQM-266 (10 μM). *p < 0.05 when comparing the effect of IQM-266 on the peak current and on the charge through K_V_4.3/DREAM channels.

Figure 2A shows the concentration-dependence of block produced by IQM-266 on K_V_4.3/DREAM channels when measured at the maximum peak current. By fitting these data to a Hill equation, the concentration that inhibited 50% of the channels (IC_50_) and the Hill coefficient (*n*) were calculated (8.6 μM and 0.75, respectively, *n* = 34). Figure 2A also shows the Hill fit in which we assume that this curve exhibits a basal value of 0 and a maximum percentage block of 100 (dashed line). The IC_50_ and the *n* obtained with this fit were very similar to those obtained without any constriction (9.0 μM and 1.0, respectively). The *n* value obtained led us to conclude that binding of IQM-266 to K_V_4.3/DREAM channels does not exhibit cooperativity. Figure 2B exhibits a bar graph in which the effects of this compound on the maximum peak current and on the charge (measured as the integral of the current recordings) are shown. At all concentrations tested, IQM-266 decreased the maximum peak current and, to a lesser extent, the charge through the membrane, these differences being very marked between 1 and 10 μM. Indeed, at 3 μM, IQM-266 decreased the maximum peak current (33.7 ± 5.3%, *n* = 6), but increased the charge (13.0 ± 4.1%, *n* = 6), which can be explained by the slowing effect on the inactivation kinetics produced by this compound (Figure 2C). IQM-266 slows down both the activation and the inactivation kinetics of the current in a concentration-dependent manner. Therefore, the equilibrium between the decrease in the maximum peak current and the slowing of the inactivation process lead, either to an increase (at 3 μM) or to a decrease in the charge (concentrations >3 μM; Figure 2D). In order to characterize both effects: the increase and the inhibition of the charge through K_V_4.3/DREAM channels, two different concentrations of IQM-266 were used, 3 and 10 μM (close to the IC_50_), respectively.

### Time Dependent Effects of IQM-266 on K_V_4.3/DREAM Channels

**Figure 3:**
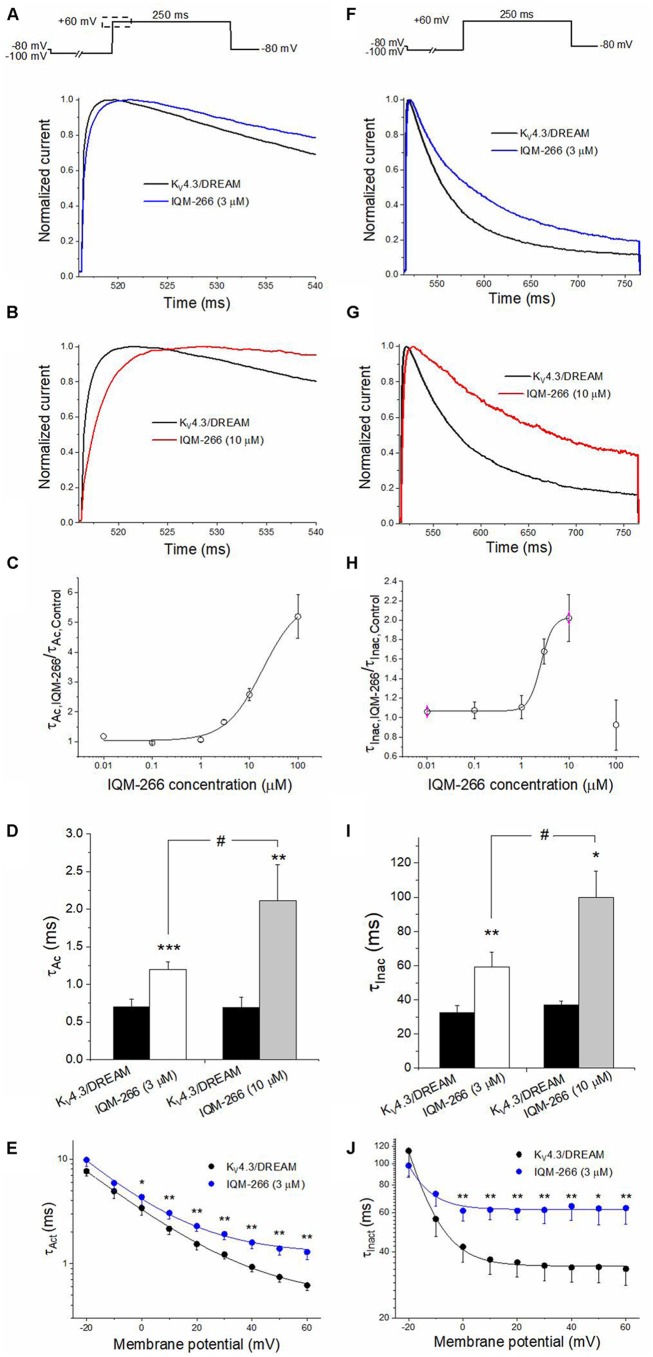
Activation and inactivation kinetics of K_V_4.3/DREAM currents in the absence and in the presence of IQM-266. **(A)** Normalized first 20 ms of original current recordings in control and in the presence of IQM-266 (3 μM). The current records were fitted to a monoexponential equation in order to obtain the activation time constant (τ_Ac_). **(B)** Normalized first 20 ms of original current recordings in control and in the presence of IQM-266 (10 μM). The current records were fitted to a monoexponential equation in order to obtain the *τ*_Ac_ values. **(C)** Concentration-dependence of the τ_Ac,IQM-266_/τ_Ac,Control_. **(D)** Histogram representing the τ_Ac_ in the different experimental conditions. **(E)** Voltage-dependent effects of IQM-266 (3 μM) on the time constant of activation (τ_Act_).**(F)** Normalized first 250 ms of original current recordings in control and in the presence of IQM-266 (3 μM). The current records were fitted to a monoexponential equation in order to obtain the inactivation time constant (τ_Inac_). **(G)** Normalized first 250 ms of original current recordings in control and in the presence of IQM-266 (10 μM). The current records were fitted to a monoexponential equation in order to obtain the *τ*_Inac_ values.**(H)** Concentration-dependence of the τ_Inac, IQM-266_/τ_Inac, Control_. **(I)** Histogram representing the τ_Inac_ in the different experimental conditions.**(J)** Voltage-dependent effects of IQM-266 (3 μM) on the time constant of inactivation (τ_Inact_). *p < 0.05, **p < 0.01 and ***p < 0.001 when comparing data in presence of IQM-266 with control; ^#^*p* < 0.05 when comparing data obtained in the presence of IQM-266 (3 μM) and IQM-266 (10 μM).

The activation kinetics of K_V_4.3/DREAM current in the absence and in the presence of IQM-266 was analyzed by fitting the traces to a monoexponential equation, from which the activation time constant (τ_Act_) was obtained. The inactivation process was also fitted to a monoexponential curve after applying a 250 ms depolarizing pulse from −80 mV to +60 mV, from which the time constant of inactivation (τ_Inac_) of the K_V_4.3/DREAM, in the absence and in the presence of IQM-266, were obtained (Figure 3). Figures 3A,B show the first 25 ms of the normalized currents after applying a depolarizing pulse from −100 to +60 mV in the absence and in the presence of 3 or 10 μM IQM-266. In order to analyze the concentration-dependence of this slowing in the activation kinetics, the τ_Ac,IQM-266_/τ_Ac, Control_ ratio vs. IQM-266 concentration was plotted (Figure 3C). As it can be observed, the slowing effect induced by this compound was concentration-dependent. Figure 3D shows the absolute values under control and in the presence of IQM-266 at 3 and 10 μM, respectively. This slowing effect on the activation kinetics was observed at membrane potentials positive to 0 mV (*p* < 0.05). This compound also slows the inactivation process at membrane potentials positive to 0 mV (*p* < 0.05), therefore, this slowing is concentration-dependent (Figures 3F–J).

### Effects of IQM-266 on the Recovery Kinetics of Inactivation of K_V_4.3/DREAM Channels

**Figure 4:**
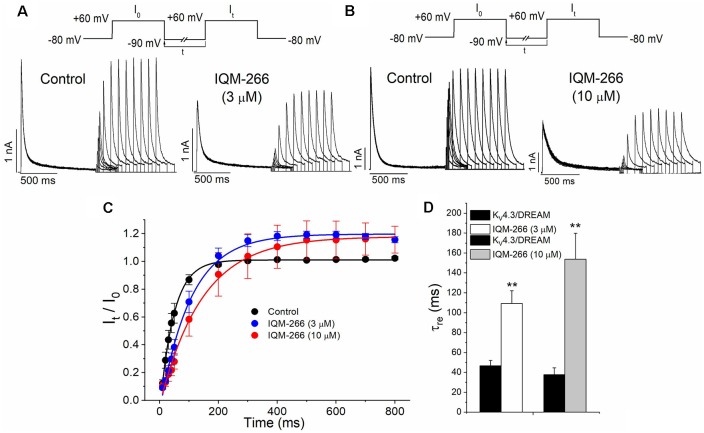
Inactivation recovery kinetics of K_V_4.3/DREAM channels in the absence and in the presence of IQM-266. Original recordings after applying the pulse protocols shown in the upper part of the figure in the absence and in the presence of IQM-266 **(A)** 3 μM or **(B)** 10 μM. **(C)** Data obtained after plotting the *I*_t_/*I*_0_ vs. the interpulse period separating both, the conditioning and the test pulse. **(D)** Histogram bar showing the time constant of recovery obtained under control and in the presence of IQM-266 (*n* = 4–5). **p < 0.01 when comparing data in presence of IQM-266 with control.

In order to analyze the recovery from inactivation of K_V_4.3/DREAM channels, a double pulse protocol was applied (Figures 4A,B, upper panel), consisting in a conditioning prepulse from −80 to +60 mV of 1 s in duration (I_0_) in order to inactivate most of the channels, followed by a test pulse applied after a variable interpulse (between 10 and 800 ms at −90 mV) to +60 mV (I_t_). This pulse protocol was applied before and after perfusing the cells with 3 μM or 10 μM IQM-266. The ratio I_t_/I_0_ measured at the maximum peak was plotted vs. the time interpulse between the end of I_0_ and the application of I_t_. In all experimental conditions, data were fitted to a monoexponential function, from which the time constant of recovery (τ_re_) was obtained. Under control conditions, the τ_re_ arose a mean value of 42.7 ± 4.5 ms (*n* = 8). IQM-266 slowed the recovery process of the K_V_4.3/DREAM current in a concentration-dependent manner (from 46.8 ± 5.3 to 109.2 ± 13.0 ms in control and in the presence of IQM-266 3 μM, *n* = 5, *p* < 0.01 and from 37.7 ± 6.9 to 153.6 ± 26.3 ms in control and in the presence of IQM-266 10 μM *n* = 4 *p* < 0.05; Figures 4C,D). Interestingly, both concentrations of IQM-266 induced an overshoot in the recovery process similar to that observed in cardiac *I*_to_, and that has been attributed to KCNE2 effects (Wettwer et al., 1993; Zhang et al., 2001; Radicke et al., 2008).

### Voltage Dependence Effects of IQM-266 on K_V_4.3/DREAM Channels

**Figure 5:**
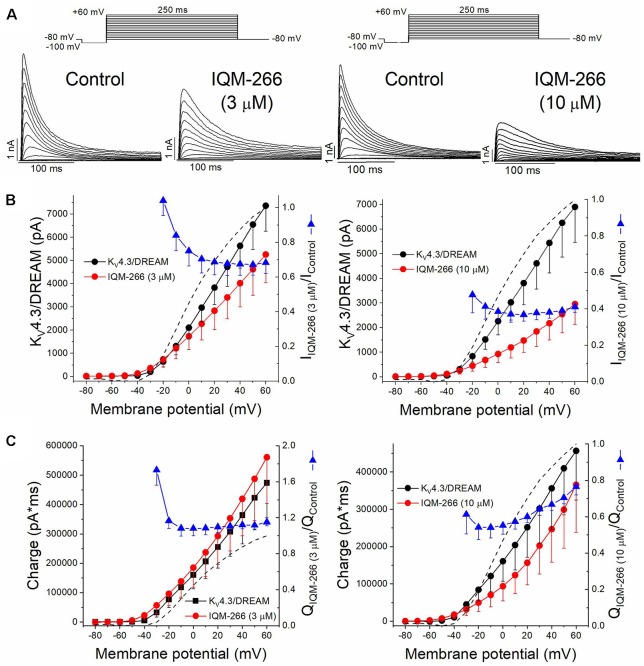
Voltage dependence interaction between IQM-266 and K_V_4.3/DREAM channels. **(A)** Original recordings obtained generated by K_V_4.3/DREAM channels in the absence and in the presence of IQM-266 after applying the pulse protocols shown in the upper part of the figure. **(B)** I-V relationship of the currents generated by K_V_4.3/DREAM channels in the absence and in the presence of IQM-266 at 3 μM (left) and 10 μM (right) when measured at the maximum peak current. It is also shown the relative current (I_IQM-266_/I_Control_) vs. membrane potential. Dotted and dashed lines show the activation and the inactivation curves, respectively (*n* = 4–6). **(C)** Q-V relationship of the charge through K_V_4.3/DREAM channels in the absence and in the presence of IQM-266 at 3 μM (left) and 10 μM (right) when measured at the area under the current during the application of depolarizing pulse protocol shown in the upper part of the figure. It is also shown the relative charge (Q_IQM-266_/Q_Control_) vs. membrane potential. Dotted and dashed lines show the activation and the inactivation curves, respectively (*n* = 4–6).

Figure 5A shows superimposed current traces obtained in the absence and in the presence of IQM-266 at 3 or 10 μM. After plotting the maximum peak current obtained under control and after perfusion with external solution containing 3 μM or 10 μM IQM-266, the current-voltage (I-V) relationships were obtained (Figure 5B). These two plots also show the ratio I_IQM-266_/I_Control_ at both concentrations (blue triangles), together with the activation curve (dashed line). A block of the maximum peak current produced by IQM-266 3 μM increased in the range of activation of K_V_4.3/DREAM channels but remained constant at membrane potentials positive to +10 mV. At higher concentrations, the maximum block was obtained at +20 mV (63.1 ± 3.6%, *n* = 4) and it decreased only when the more positive potential was applied (58.8 ± 3.0% at +60 mV, *n* = 4, *p* < 0.05). Figure 5C shows the charge-voltage relationships (Q-V) obtained after plotting the charge under the current obtained in control and in the presence of IQM-266 3 or 10 μM. These plots also show the relative charge (blue triangles) at each membrane potential in order to analyze the voltage dependence of block. IQM-266 at 3 μM increased the charge in a voltage independent manner. The maximum block (measured at the charge) produced by IQM-266 at 10 μM was observed at −20 mV and this block decreased in a voltage dependent manner (45.8 ± 6.7% vs. 23.5 ± 4.5% at −20 mV and +60 mV, respectively, *n* = 4, *p* < 0.05). Importantly; both the increase of the charge and the decrease in the maximum peak current were observed at all membrane potentials.

The activation curves were obtained from the I-V relationships. Data were plotted against membrane potential to which each current record was generated and fitted to a Boltzmann equation, in order to obtain the *V*_h_ and *s* values. IQM-266, at 3 μM, did not shift the activation curve (*V*_h_ = +4.3 ± 2.6 mV and +7.8 ± 3.9 mV in the absence and in the presence of IQM-266, *n* = 5, *p* > 0.05; *s* = 16.1 ± 0.3 mV and 19.6 ± 0.6 mV, *n* = 5, *p* > 0.05).

**Figure 6:**
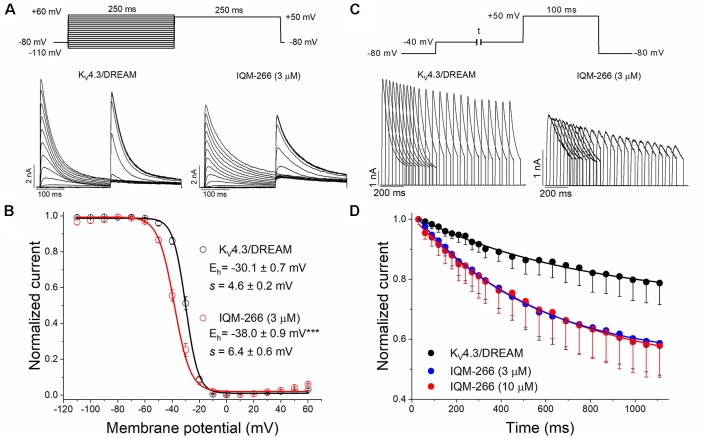
Effects of IQM-266 on the steady-state inactivation curve and on the closed-state inactivation. **(A)** Original recordings obtained generated by K_V_4.3/DREAM channels in the absence and in the presence of IQM-266 after applying the pulse protocols shown in the upper part of the figure. **(B)** Steady-state inactivation in cells expressing K_V_4.3/DREAM channels in the absence and in the presence of IQM-266 (3 μM) are shown. Peak currents in response to test step to +50 mV after various conditioning potentials (*V*_h_) are shown. **(C)** Original recordings recorded from K_V_4.3/DREAM channels after applying the pulse protocol shown in the upper part of the figure generated by in the absence and in the presence of IQM-266 (3 μM). **(D)** Development of closed-state inactivation of K_V_4.3/DREAM channels at a membrane potential (−40 mV) where this current inactivates, but does not conduct. Note how IQM-266 increases the closed-state inactivation.

In order to study the voltage dependence of inactivation of K_V_4.3/DREAM channels, a double pulse protocol consisting in a 250 ms conditioning pulse to different potentials between −110 and +60 mV, followed by a test pulse to +50 mV of 250 ms in duration, was applied (Figure 6A). The maximum peak currents measured at the test pulse were plotted against the membrane potential of the previous conditioning pulse, and the data were fitted to a Boltzmann equation. IQM-266, at 3 μM, shifted the inactivation curve to negative potentials (*V*_h_ = −30.1 ± 0.7 mV and −38.0 ± 0.9 mV in the absence and in the presence of IQM-266, *n* = 4, *p* < 0.01; *s* = 4.5 ± 0.2 mV and 6.6 ± 0.7 mV, *n* = 4, *p* > 0.05; Figure 6C).

Since K_V_4.3 channels inactivate predominantly from the closed state (Campbell et al., 1993; Beck and Covarrubias, 2001), the negative shift induced by IQM-266 of the steady-state inactivation curve is indicative of an acceleration of the closed-state inactivation. In order to elucidate this issue, a double pulse protocol was applied. A pre-pulse test to +60 mV during 100 ms was immediately preceded by a pulse to −40 mV of variable duration (Figure 6B). As it can be observed, IQM-266 increased the degree of closed-state inactivated channels, thus suggesting that IQM-266 promotes inactivation from the closed-state (Figure 6D).

### Effects of IQM-266 on K_V_4.3 Channels

**Figure 7:**
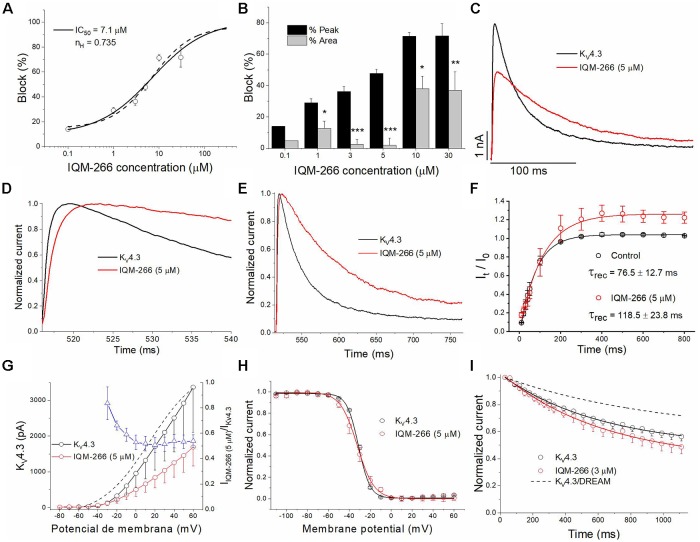
Effects of IQM-266 on K_V_4.3 channels. **(A)** Concentration-block of K_V_4.3 channels induced by IQM-266. The IC_50_ obtained after fitting the data points resulted to be 7.1 μM. **(B)** Percentage of block of K_V_4.3 channels measuring the peak current and the charge. **(C)** Current recordings from −80 to +60 mV in the absence and in the presence of IQM-266 (5 μM). **(D)** First 20 ms of original current recordings from −80 to +60 mV in control and in the presence of IQM-266 (5 μM). **(E)** First 250 ms of original current recordings from −80 to +60 mV in control and in the presence of IQM-266 (5 μM). **(F)** Recovery kinetics from inactivation in the absence and in the presence of IQM-266 (5 μM; *n* = 4). Note that this compound does not modifies this process. **(G)** I-V relationship of the currents generated by K_V_4.3 channels in the absence and in the presence of IQM-266 (5 μM) when measured at the maximum peak current. It is also shown the relative current (I_IQM-266_/I_Control_) vs. membrane potential. Dashed line shows the activation curve (*n* = 6). **(H)** Steady-state inactivation curve obtained in the same manner than shown in Figure 6 (*n* = 4). **(I)** Development of the closed-state inactivation of K_V_4.3 channels at a membrane potential (−40 mV) where this current inactivates, but does not conduct. Note that IQM-266 does not modify the closed-state inactivation. *p < 0.05, **p < 0.01 and ***p < 0.001 when comparing data in presence of IQM-266 with control.

In order to analyze the selectivity of IQM-266 for K_V_4.3/DREAM over K_V_4.3 channels, the effects of IQM-266 were studied on K_V_4.3 channels expressed in CHO cells in the absence of DREAM. As it is shown in the Figure 7, the *IC*_50_ value obtained when measured at the maximum peak current was very close to that observed in K_V_4.3/DREAM channels (7.1 μM vs. 8.6 μM, *n* = 21–23). Also, IQM-266 slowed the activation kinetics (*τ* = 0.73 ± 0.09 ms vs. 1.92 ± 0.32 ms, in control and in the presence of IQM-266, respectively, *n* = 8, *p* < 0.01), as well as the inactivation kinetics. In fact, the latter process that exhibits a biexponential decay under control conditions (*τ*_f_ = 19.6 ± 2.4 ms and *τ*_s_ = 76.2 ± 9.6 ms, *n* = 14) becomes monoexponential in the presence of IQM-266 (*τ* = 62.3 ± 4.7 ms, *n* = 14, *p* > 0.05 vs. *τ*_s_ value in control conditions; Figures 7C–E). However, IQM-266 did not increase the charge at any concentration tested (Figure 7B). In contrast to what occurs in the presence of DREAM, IQM-266 did not modify the recovery kinetics of inactivation (Figure 7F). Figure 7G shows the two I-V relationships obtained in the absence and in the presence of IQM-266, together with the ratio I_IQM-266_/I_Control_ (blue triangles) and the activation curve (dashed line). A block of the maximum peak current produced by IQM-266 increased in the range of activation of K_V_4.3 channels but remained constant at membrane potentials positive to +10 mV. IQM-266 did not shift the inactivation curve to more negative potentials (Figure 7H), and it did not modify the closed-state inactivation (Figures 7H,I). All these results indicate that, although this compound also binds to K_V_4.3 channels, the increase in the charge observed in K_V_4.3/DREAM channels and induced by IQM-266 is due to its specific interaction with DREAM. Moreover, this interaction seems to prevent the effect of DREAM on the recovery from inactivation.

### Effect of IQM-266 on *I*_A_ From DRG Neurons

**Figure 8:**
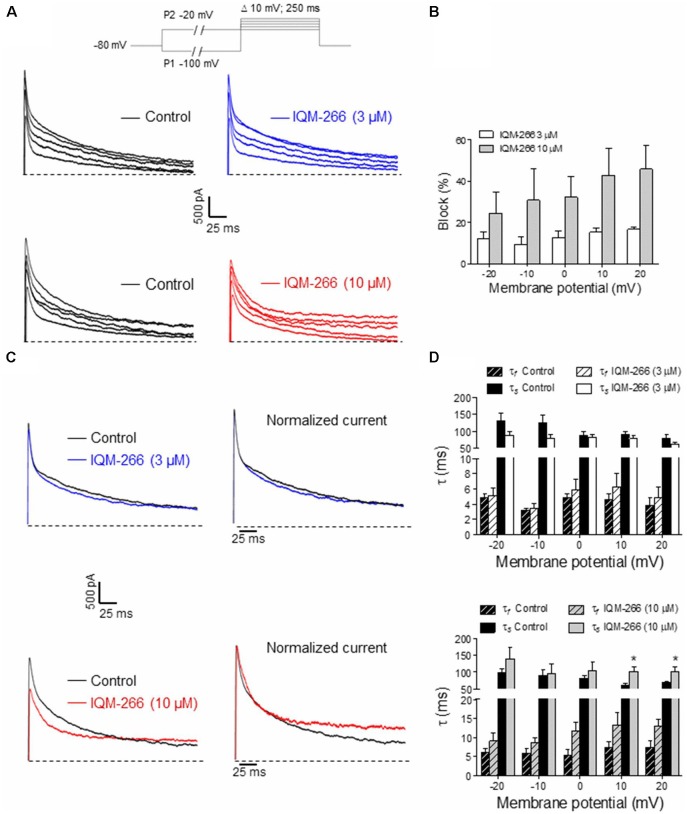
Effects of IQM-266 on peak amplitude and inactivation kinetics of *I*_A_ from dorsal root ganglia (DRG) neurons. **(A)** Representative recordings of *I*_A_ isolated by using the voltage protocol depicted on top of the panels (see “Materials and Methods” section). *Upper panels* show original *I*_A_ recordings in the absence (Control) and the presence of IQM-266 (3 μM); *lower panels* show *I*_A_ recordings in the absence (Control) and the presence of IQM-266 (10 μM). **(B)** Bar graph of percent block of peak *I*_A_ by IQM-266 (3 μM or 10 μM) at different potentials. Data were obtained from eight cells for IQM-266 (3 μM), and five cells for IQM-266 (10 μM). **(C)** Effect of IQM-266 (3 μM; upper panels) and (10 μM; lower panels) on inactivation kinetics of I_A_. Original recordings (*left panels*) have been normalized to peak *I*_A_ in the absence of IQM-2666 (*right panels*) to better appreciate the change in inactivation kinetics. **(D)** Bar graph showing the effect of IQM-266 at 3 μM (upper graph) or 10 μM on time constants of inactivation at different potentials. The current records were fitted to a biexponential equation to obtain the *τ*_f_ and *τ*_s_ values. Data are from eight cells for IQM-266 (3 μM), and five cells for IQM-266 (10 μM). *p < 0.05 with regard to Control.

DRG neurons are known to express DREAM as well as K_V_4.3 channels, which contribute to *I*_A_ (Phuket and Covarrubias, 2009; Tsantoulas and McMahon, 2014; Tian et al., 2018). Hence, we decided to record *I*_A_ from rat DRG neurons in order to evaluate the effect of IQM-266 on native potassium channels. By using a voltage protocol designed to isolate *I*_A_, we recorded transient, fast activating and inactivating potassium currents in the voltage-range in which *I*_A_ makes a substantial contribution to voltage-dependent potassium currents (−20 mV to +20 mV) (Figure 8A). Isolated currents were sensitive to 4-aminopyridine 5 mM (data not shown) and displayed inactivation kinetics that required the sum of two exponential terms for an adequate description. Time constant values and relative amplitude of the fast and slow kinetic components at 0 mV were *τ*_f_ = 5.1 ± 0.6 ms (45%) and *τ*_s_ = 86.1 ± 7.5 ms (55%, *n* = 13 cells). As previously reported, time constants exhibited weak voltage-dependence (Figure 8B), and the relative weight of the two components barely changed in the voltage range that we studied (data not shown; Phuket and Covarrubias, 2009).

Interestingly, IQM-266 at 3 and 10 μM reduced the peak amplitude of *I*_A_ dose dependently. Percentage block showed slight voltage dependence, increasing with the depolarization (Figures 8A,B). Likewise, IQM-266 at 10 μM slowed current inactivation by increasing both τ_f_ and τ_s_. This effect reached statistical significance on τ_s_ and developed in a voltage-dependent manner (at potentials equal and positive to 0 mV; Figures 8C,D).

## Discussion

In the present study the effects of IQM-266 on recombinant K_V_4.3 and K_V_4.3/DREAM channels expressed in mammalian cells, as well as on *I*_A_ from DRG neurons, have been analyzed. We demonstrate that this new compound: (1) binds to K_V_4.3/DREAM channels in a concentration-, time- and voltage-dependent manner; (2) inhibits the maximum peak current and, to a lesser extent, the charge crossing the cell membrane during depolarization; (3) at certain concentrations (3 μM), IQM-266 increases the charge through the cell membrane during the application of depolarizing pulses; and (4) inhibits peak *I*_A_ amplitude while slowing its inactivation. Overall, the results presented here are consistent with a preferential binding of IQM-266 to a pre-activated closed state of K_V_4.3/DREAM channels.

In the last decade, there small molecules have been developed that bind to DREAM and modify K_V_4 channel function (Gonzalez et al., 2014; Naranjo et al., 2016). Among them, repaglinide and CL-888 were shown to inhibit *I*_A_, whereas NS5806 would be the only DREAM ligand able to potentiate this sort of potassium current (Witzel et al., 2012; Gonzalez et al., 2014). Like IQM-266, NS5806 slowed down the inactivation decay of neuronal *I*_A_ and slightly decreased the maximum peak current.

The two main reported effects of DREAM on K_V_4.3 channels are: (i) an increased traffic of K_V_4.3 channels to the membrane; and (ii) an acceleration of the activation and recovery kinetics from inactivation (An et al., 2000; Naranjo et al., 2016). In the present study, we show that IQM-266 produces the opposite effects to those induced by DREAM: slowing of the activation and recovery from inactivation kinetics, which might be attributed to IQM-266 binding to DREAM, hence supporting the results of the SPR experiments. IQM-266 interacts with K_V_4.3/DREAM channels in a concentration-, time- and voltage-dependent manner, consistent with binding preferentially to a pre-activated closed state of the channels and with very low or no affinity for the open state. There are several pieces of evidence supporting this mechanism of action: (1) the maximum degree of block (produced by 10 μM IQM-266, a concentration close to its IC_50_) measured at the maximum peak and at the charge, was obtained at −10 mV, a potential at which most of the channels are closed or in pre-activated, closed states; (2) block steeply increased in the activation range of K_V_4.3/DREAM channels, achieving a maximum and plateau level at potentials positive to +20 mV, where the probability of channels opening increases; (3) IQM-266 slows the activation of the K_V_4.3/DREAM current. Also, IQM-266 slows the recovery process from inactivation, suggesting that its binding to K_V_4.3/DREAM channels promotes inactivation; and (4) in fact, IQM-266 negatively shifted the inactivation curve, as well as the closed-state inactivation. Since K_V_4.3 channels mostly inactivate from the closed states (Beck and Covarrubias, 2001), this result is consistent with the interaction of IQM-266 with a closed or pre-activated closed state of the channels (Snyders et al., 1992; Longobardo et al., 1998).

Furthermore, IQM-266 inhibits K_V_4.3 current in the absence of DREAM. However, the K_V_4.3 IQM-266 interaction exhibits differential features. In the presence of DREAM, IQM-266 prevents the effect of this regulatory subunit on the channel (acceleration of: (i) recovery from inactivation and activation; (ii) slower decay kinetics; and (iii) less prominent closed-state inactivation), yet in the absence of DREAM, IQM-266 decreased the peak potassium current and slowed the activation and the inactivation kinetics.

However, the more striking effect produced by IQM-266 is the slowing of inactivation kinetics. Indeed, this effect may explain why at concentrations lower than the IC_50_, IQM-266 augments the efflux of potassium ions resulting in an increase in charge (activating effect). Importantly, this increase in the charge is observed at membrane potentials positive to +10 mV. This effect is more evident at concentrations at which the inhibition of the maximum peak current is negligible, but still capable of slowing the inactivation decay. This effect could be the basis of a promising therapeutic strategy for the treatment of certain pathologies affecting cardiac (cardiac arrhythmias) or neuronal (epilepsy, Alzheimer disease or ataxia) cells, in which a downregulation of K_V_4.3 or DREAM has been demonstrated (Huo et al., 2014; Hall et al., 2015; Smets et al., 2015; Villa and Combi, 2016). IQM-266 also modulated *I*_A_ from rat DRG neurons. At 10 μM, IQM-266 effects on *I*_A_ were reminiscent of those observed on heterologously expressed K_V_4.3/DREAM channels. Hence, IQM-266 10 μM inhibited peak *I*_A_, and this effect increased with the depolarization in the physiological range of activation of the current. Likewise, IQM-266 10 μM slowed inactivation kinetics at potentials positive to 0 mV. In contrast, no facilitation of *I*_A_ could be observed with IQM-266 3 μM. At present, we do not have an explanation for this result except the fact that DRG neurons express other potassium channel regulatory proteins in addition to DREAM (David et al., 2012; Cheng et al., 2016; Tian et al., 2018), which may prevent the potentiating effect seen at 3 μM IQM-266 on K_V_4.3/DREAM channels. Notwithstanding, our results in DRG neurons suggest that IQM-266 constitutes a small, novel chemical molecule suitable to modulate K_V_4.3 channels in native systems.

Different neuronal (Hall et al., 2015; Gross et al., 2016) or cardiac pathologies are related to abnormalities in the function of different ion channels and/or regulatory subunits, such as K_V_4.3 and the regulatory subunit DREAM. Thus, KChIPs starts to emerge as a realistic drug target, and IQM-266 could be considered as a new chemical tool that might allow a better understanding of: (i) DREAM physiological role; and (ii) the modulation of *I*_A_ in pathological conditions.

## Author Contributions

DP, PC, LL, PM, YM, PS, CIG, SS and AL-H performed the experiments and analyzed the data. MM-M, LO-O, AA, JN, MG-R and CV conceived the study, analyzed the data and wrote the article.

## Conflict of Interest Statement

The authors declare that the research was conducted in the absence of any commercial or financial relationships that could be construed as a potential conflict of interest.
